# The relationship between pond habitat depth and functional tadpole diversity in an agricultural landscape

**DOI:** 10.1098/rsos.150165

**Published:** 2015-07-22

**Authors:** Cássia de Souza Queiroz, Fernando Rodrigues da Silva, Denise de Cerqueira Rossa-Feres

**Affiliations:** 1 Programa de Pos-Graduação em Biologia Animal, Universidade Estadual Paulista – UNESP, Campus de São José do Rio Preto, São José do Rio Preto, SP, Brazil; 2 Departamento de Zoologia e Botânica, Universidade Estadual Paulista – UNESP, Campus de São José do Rio Preto, São José do Rio Preto, SP, Brazil; 3 Departamento de Ciências Ambientais, Universidade Federal de São Carlos, Campus Sorocaba, Rodovia João Leme dos Santos Km 110, Sorocaba, SP, Brazil

**Keywords:** anuran, land use, biodiversity loss, pasture matrix, traits

## Abstract

One of the most important goals of biodiversity studies is to identify which characteristics of local habitats act as filters that determine the diversity of functional traits along environmental gradients. In this study, we investigated the relationship between the environmental variables of ponds and the functional trait diversity distribution of anuran tadpoles in an agricultural area in southeastern Brazil. Our results show that the functional trait diversity of frog tadpoles has a bell-curve-shaped relationship with the depths of ponds inserted in a pasture matrix. Because we are witnessing increasing human pressure on land use, simple acts (e.g. maintaining reproductive habitats with medium depth) can be the first steps towards preserving the diversity of Neotropical frog tadpole traits in agricultural landscapes.

## 1. Introduction

A fundamental concept in community assembly theory is that when a regional species pool (e.g. the species group likely to colonize a local community [1]) is recognized, it is possible to predict which biotic (e.g. the presence of predators) and abiotic (e.g. climatic variables) characteristics act as filters to select the composition of species in local habitats [2–4]. In this context, habitat characteristics act as one of the selective forces on functional species traits, influencing the species composition of communities on a local scale [2,3,5]. Functional traits are defined as any phenotypic character that indirectly affects the fitness of the organism through biochemical, physiological, morphological, developmental or behavioural mechanisms [6]. The potential of a species to establish itself and persist under any set of environmental conditions is largely influenced by the biological characteristics of the species [2,7,8]. Therefore, identifying how the characteristics of local habitats are related to functional diversity may bring new insight into the mechanisms that determine the assembly of communities in modified landscapes.

In this paper, we investigated the relationship between the functional diversity of frog tadpoles and the environmental descriptors of 38 ponds in an agricultural area in southeastern Brazil that consisted primarily of a matrix of pastures. Although recent studies in this region have shown a strong influence of hydroperiod on the richness of species in adult anuran communities [9,10], the depth of ponds may represent more accurate habitat information for habitat use by tadpoles because they present different swimming traits (i.e. nektonic or benthic). For example, permanent reproductive habitats can be represented by puddles or ponds displaying great depth, as these habitats can be represented by shallow marshes. Furthermore, the increasing complexity of vegetation in ponds is an important factor in explaining the taxonomic diversity in tadpole communities [11,12]. However, with the expansion of agriculture [13], ponds are becoming more homogeneous because of the absence or reduction of vegetation cover in breeding habitats, and its effects on tadpole functional trait diversity are still unknown. Therefore, we are attempting to understand how the characteristics of ponds in an agricultural area are related to the functional diversity in the larval stage of frogs. Our predictions are the following: (i) given that the characteristics of ponds can act as a filter for the occurrence of tadpoles of some species, we predict that shallow reproductive habitats will harbour less functional diversity than deep ones; and/or (ii) because the homogenization of ponds can limit the diversity of functional traits, we predict that reproductive habitats with a higher number of vegetation types (increasing feeding habits and providing refuge from predators) will harbour higher functional diversity than reproductive habitats with fewer vegetation types. We hope that an understanding of how the functional spaces occupied by tadpole communities change along these gradients will be useful in anticipating the potential loss of trait diversity that is associated with biodiversity erosion in altered landscapes.

## 2. Material and methods

### 2.1 Data acquisition

To test our hypotheses, we gathered information on the species composition of frog tadpoles in 38 ponds from four previous studies performed by our laboratory [9,14–16]. Because we obtained all data from literature surveys, no specific permission or licence to conduct the fieldwork was required. The four studies used the same tadpoles and pond characteristic sampling methodologies and carried out the surveys of tadpoles during one year. These studies examined the association of environmental descriptors of ponds on species richness and anuran, but we knew nothing of the relationship of these descriptors with frog tadpole traits. The region where the studies were developed was originally covered with semi-deciduous forest and patches of Cerrado biome, which were altered during the establishment of agricultural crops. Currently, this region is considered one of the most deforested and fragmented in the state [13].

Frog tadpoles were collected in 38 ponds with different physiognomic characteristics. All ponds were located in a pasture matrix and were at least 1000 m away from sugarcane, orange and rubber plantations. For each of the 25 frog species recorded, we compiled 11 functional traits of tadpoles (table 1) for five tadpoles between stages 33 and 39 (*sensu* Gosner 1960) of each species. The tadpoles measured are deposited in DZSJRP—Amphibian Tadpole Collection of Department of Zoology and Botany, UNESP, São José do Rio Preto. The traits were chosen because they have well-known relationships with tadpole feeding and swimming behaviours, habitat use or life-history strategies [17–21]. To test our hypotheses, we compiled two environmental descriptors for the 38 ponds: (i) the maximum depth (DEPTH) of each breeding habitat, which ranged from 0.1 to 2.1 m (an average depth of 0.7 m); and (ii) the number of vegetation types in the interior of ponds (NVI), scored as one of four categories of increasing complexity.

**Table 1.** Traits used to measure tadpole functional diversity. To determine the morphometric measurements (i.e. continuous variables), we used the average of five individuals between stages 33 and 39 for each species (*sensu* Gosner 1960).

trait type	trait	variables
position on the water column	benthic (live in the bottom of ponds, either in shallow or deep water), nektonic (live in open water of ponds, often moving through vegetation) or neustonic (move from bottom to surface films of ponds to feed on organisms)	categorical
feeding behaviour	scratcher (feeding by rasping substrate or taking in particulate matter), filter (feeding by filtering microscopic particles out of the water) or macrophagous (feeding on large food particles)	categorical
position of the eyes	lateral or dorsal	categorical
position of oral disc	quantified by the angular orientation of the oral disc of anuran tadpoles relative to a defined longitudinal body axis [15]: terminal (90° angle—extreme one), ventral (0° angle—extreme one) or antero-ventral (angles that fall between the two previous positions)	categorical
presence of flagella	presence or absence	binary
body form	body length/total length	continuous
body form	body width/total length	continuous
body form	body height/total length	continuous
body form	width of the tail musculature/total length	continuous
body form	height of dorsal tail/width of the tail musculature	continuous
body form	height of ventral tail/width of the tail musculature	continuous

### 2.2 Data analysis

We computed the functional dispersion (FDis), which is a multi-dimensional index based on multi-trait dispersion [22]. It measures the mean distance of an individual species to the centroid of all species in the community [22]. FDis has no upper limit, and small values indicate that communities are composed of species with similar traits, whereas high values indicate that communities are composed of species with distinct traits. According to Laliberté & Legendre [22], this index presents several desirable properties: (i) it is by construction unaffected by species richness; (ii) it can be computed from any distance or dissimilarity measure; (iii) it can handle any number and type of traits (including more traits than species); and (iv) it is not strongly influenced by outliers.

To reduce dimensionality and correlations between continuous variables within our trait database, we performed a principal component analysis (PCA) on measures of tadpole body forms (table 1). The first two principal component axes explained 75% of the variation of these measures (see results in electronic supplementary material, appendix S1). Therefore, for subsequent analysis, we used the first two axes of PCA along with categorical traits (see electronic supplementary material, appendix S1). We used Gower's distance to measure the differences in trait variation across species because it accommodates quantitative, nominal and categorical variables in a single measure [23]. Then, following previous studies [24], we used generalized least-squares models with different combinations of predictor variables taking spatial autocorrelation (i.e. locations close to each other exhibit more similar values than those further apart) into account by fitting an exponential spatial covariance structure [25]. To determine which model best described the FDis values, we used the Akaike information criterion, corrected for the small sample size (AICc) [26]. In addition, to evaluate the uncertainty of the model selection, we used the weight of the model AICc (wAICc) that expressed the weight of the evidence favouring the model as the best among all models compared [26].

To permit comparative analyses among ponds, we tested whether observed FDis values for each pond were higher or lower than simulated values, using a null model in which species richness was fixed and only the identities of the species in the ponds were randomized 999 times. To obtain a significance test, we computed the observed value ranks in the null distribution, and then we calculated a *p*-value by dividing it by the number of null model interactions plus one [27]. All analyses were performed in R software [28] using the functions in FD [29], Picante [30] and bbmle [31] packages.

## 3. Results

**Figure 1.**
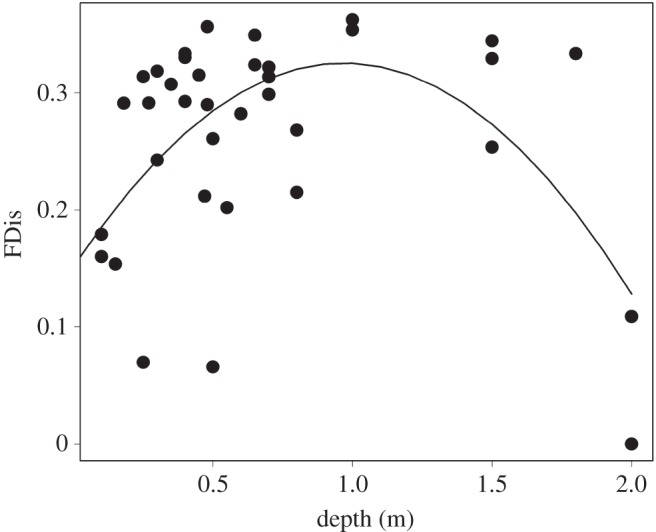
Relationship between FDis values and the depths of 38 ponds in an agricultural area in southeastern Brazil.

**Figure 2.**
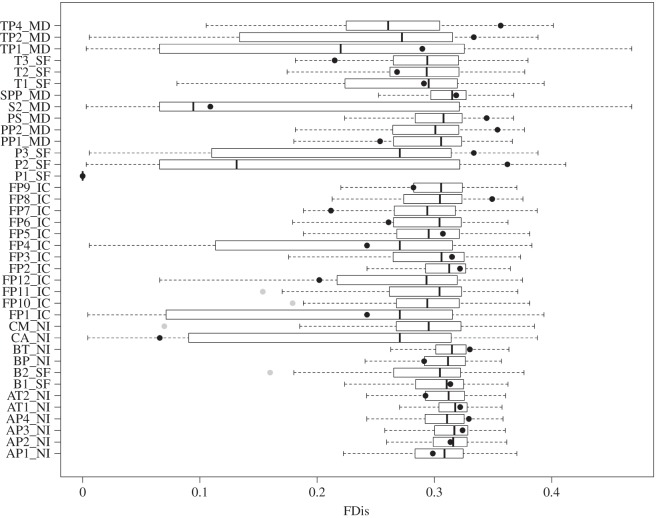
Boxplot showing 999 randomized FDis values for each pond. Circles are observed FDis values. Grey circles represent *p*<0.05, whereas black circles represent *p*>0.05. Nomenclature of the ponds is the same as used in the original articles.

We detected that the model with a bell-curved association between FDis values and depth gradient of ponds (*p*<0.001, figure 1) was the most parsimonious (table 2). Some FDis values (10%) were lower than expected by the null expectation (figure 2), indicating that some species do not occur in ponds with extreme gradients (e.g. shallow and deep depth) probably because their traits are poorly adapted to these scenarios.

**Table 2.** Generalized least-squares models predicting the relationship between FDis values and the environmental variables of ponds. DEPTH, maximum depth of ponds; NVI, number of vegetation types in the interior of ponds; AICc, Akaike information criterion, corrected for the small sample size; Δ AICc, difference in Akaike's information criterion; *ω*, Akaike weights to evaluate model selection uncertainty; NULL, model without predictor variable (considering only intercept). Significant results (*p*≤0.05) are italicized.

	AICc	ΔAICc	*ω*AICc	*p*
DEPTH (quadratic)	−63.93	0	0.49	<*0.001*
NULL	−63.92	0.1	0.48	—
DEPTH (linear)	−56.61	7.3	0.01	>0.05
NVI (linear)	−55.47	8.5	0.007	>0.05
NVI (quadratic)	−49.44	14.5	<0.001	>0.05
DEPTH+NVI	−47.93	16.0	<0.001	>0.05

## 4. Discussion

Our results showed that ponds with intermediate depth harboured higher functional trait diversity (FDis) than ponds with extreme depths (e.g. shallow or deep depth). Thus, extreme gradients not only may alter total species richness [32] but also can cause a shift in functional space occupation by filtering species with traits that are poorly adapted to these scenarios [33]. We observed that ponds with shallow and deep depths each harboured a set of species with similar traits, whereas ponds with intermediate depths harboured species with distinct traits. Wellborn *et al*. [34] highlighted that tadpoles of some species are not found in either short-hydroperiod ponds (i.e. shallow depth) because of their high risk of desiccation, or permanent-water ponds (i.e. deep depth) because of their elevated number of predators. We observed that the low functional trait diversity in shallow and deep ponds is caused by the low occurrence of treefrog tadpoles (i.e. species from the genera *Dendropsophus*, *Scinax* and *Trachycephalus*) with traits associated with midwater dwelling. These treefrog tadpoles occurred predominantly in ponds having intermediate depths. Therefore, tadpoles with triangular bodies, high dorsal and ventral fins, and the presence of flagella may have their performance enhanced in intermediate ponds. For example, kinematic studies of both fish and frog tadpoles suggest that traits associated with fins and tails improve swimming [19,35–37]. Furthermore, these nektonic tadpoles are susceptible to fish predation [38]. Thus, shallow and deep depths may act as filters for some frog tadpole traits, contributing to this discrepancy in functional diversity along a depth gradient.

Currently, ecologists are increasingly emphasizing the need to predict how community and ecosystem function will respond to rapid environmental change [39,40]. Although it is widely recognized that small reproductive habitats are important for the maintenance of aquatic and semi-aquatic organismal biodiversity, they remain ignored, no matter what the outcome [41]. Our results show that the depth gradient of ponds inserted in a pasture matrix has a bell-curve-shaped association with frog tadpole functional diversity. However, we still do not know how the expansion of agriculture, currently represented mostly by sugarcane, and the homogenization of reproductive habitats affect the diversity of functional traits in the long term. Because we are witnessing increasing human pressure on land use, simple acts (e.g. maintaining reproductive habitats with medium depth) can be the first step in guiding us to protect the diversity of Neotropical frog tadpole traits in agricultural landscapes. This recommendation becomes even more important because regulations to protect small reproductive habitats are absent and overlooked in Brazilian legislation.

## Supplementary Material

Loadings of principal components analysis considering measures of tadpole body forms.

## Data accessibility

The datasets supporting this article can be accessed at the Dryad Repository (http://dx.doi.org/10.5061/dryad.16q12).
