# Supplementary material for: The relationship between pond habitat depth and functional tadpole diversity in an agricultural landscape
Source: R Soc Open Sci. 2015 Jul 22;2(7):150165. doi: 10.1098/rsos.150165 (PMC4632587; doi:10.1098/rsos.150165)
Supplement: Loadings of principal components analysis considering measures of tadpole body forms. [file rsos150165supp1.doc]

TABLE S1 – Loadings of principal components analysis considering measures of tadpole body forms.

|  | **PC1** | **PC2** |
| --- | --- | --- |
| *Dendropsophus elianeae* | -2.80353 | 0.838463 |
| *Dendropsophus minutus* | 1.077395 | 3.000002 |
| *Dendropsophus nanus* | -3.02395 | 0.994033 |
| *Dermatonotus muelleri* | 1.601129 | -0.13486 |
| *Elachistocleis bicolor* | 0.220703 | -0.33958 |
| *Elachistocleis cesarii* | -0.10902 | -1.57093 |
| *Hypsiboas albopunctatus* | -1.01458 | 0.220746 |
| *Hypsiboas faber* | -0.26206 | 0.614196 |
| *Hypsiboas raniceps* | -1.20354 | -0.19867 |
| *Leptodactylus fuscus* | 0.184467 | -0.46312 |
| *Leptodactylus labyrinthicus* | 0.23366 | -0.7094 |
| *Leptodactylus latrans* | -3.49655 | -0.73109 |
| *Leptodactylus podicipinus* | 0.272972 | -0.98709 |
| *Physalaemus centralis* | 0.554428 | -1.06938 |
| *Physalaemus cuvieri* | 0.37894 | -1.30412 |
| *Physalaemus fuscumaculatus* | 0.033347 | -0.1114 |
| *Physalaemus nattereri* | 0.804466 | -0.71531 |
| *Pseudopaludicola falcipes* | -0.38383 | 0.074462 |
| *Pseudopaludicola ternetzi* | -1.43995 | -1.77125 |
| *Rhiella schneideri* | 2.400483 | -1.86992 |
| *Rhinella ornata* | 2.895991 | -1.55649 |
| *Scinax fuscomarginatus* | -1.21597 | 1.101957 |
| *Scinax fuscovarius* | 1.531061 | 3.207288 |
| *Scinax similis* | 1.453288 | 2.531807 |
| *Trachycephalus thyphonius* | 1.310661 | 0.949655 |
| % variance explained | 0.42 | 0.75 |
